# Generative AI-Enhanced Microcalcification Detection in Full-Field Digital Mammography: Reducing False Positives with High Sensitivity

**DOI:** 10.21203/rs.3.rs-7839897/v1

**Published:** 2025-11-27

**Authors:** Kyungsu Kim, Manisha Bahl, Adham Mahmoud Alkhadrawi, Young-Tak Kim, Synho Do

**Affiliations:** 1 School of Transdisciplinary Innovations, Interdisciplinary Programs in Artificial Intelligence & Biomedical Engineering, Department of Biomedical Science, Seoul National University, Seoul, Republic of Korea; 2 Department of Radiology, Massachusetts General Brigham and Harvard Medical School, Boston, MA, USA; 3 Department of Molecular Biosciences & Bioengineering, University of Hawaii, Honolulu, HI, USA; 2 KU-KIST Graduate School of Converging Science and Technology, Korea University, Seoul, Korea; 3 Kempner Institute, Harvard University, USA, Boston, MA, USA

**Keywords:** Generative AI, Unsupervised Anomaly Detection, Refinement, Full-Field Digital Mammography, Microcalcification

## Abstract

While generative AI shows promise across industries, its advantages over state-of-the-art segmentation AI in radiology remain unclear. This study addresses this gap by developing a generative AI model to refine calcification detection in full-field digital mammograms (FFDM), improving specificity while maintaining high sensitivity. A segmentation AI initially extracted calcification pixels, achieving 98.0% sensitivity but a low positive predictive value (PPV) of 3.2%. To enhance detection, our generative AI used the segmentation AI output as a structural prior, transforming calcification-positive pixels into calcification-free pixels and generating a corrected result by subtraction. Trained on true calcification-free regions, it categorized densities within and around each calcification as interior or exterior. Our approach improved PPV by 2.28-fold (from 3.2% to 7.3%), surpassing prior generative AI models by 146-fold (from 0.05% to 7.3%), while maintaining sensitivity above 95%. It also reduced patient-level detection errors for small calcifications (5.17-fold, from 27.43% to 5.31%), high-exterior density (4.20-fold, from 53.85% to 12.82%), and low-interior density (2.89-fold, from 63.95% to 22.09%). This study serves as a seminal reference, demonstrating generative AI’s radiological significance beyond the latest segmentation models, with potential to redefine screening accuracy and generate high-fidelity virtual normal FFDM references.

## Introduction

Microcalcification detection (CD) with high sensitivity in full-field digital mammography (FFDM) is crucial for reducing breast cancer mortality [1]. Microcalcifications, tiny calcium deposits, indicate early breast cancer, particularly ductal carcinoma in situ (Stage 0) [2]. Their patterns are key markers for metastatic breast cancer, a leading cause of death in women [3]. Accurate detection is essential for preventing metastasis, enabling early intervention [4], improving prognosis, increasing survival rates, and allowing less aggressive treatments [5]. However, detecting microcalcifications is challenging, especially with small objects and dense breast tissue, making high sensitivity detection a persistent technical challenge [6]. Digital mammographic imaging screening trials emphasize this importance [7]. Accurate detection minimizes radiologists’ false negatives, ensuring early breast cancer cases aren’t missed, maintaining patient trust and screening program efficacy [8].

To enhance radiologists’ FFDM screening accuracy, technology for automatic, precise pixel-level calcification detection in FFDM is crucial. Conventional signal processing (SP) technologies like Gaussian blur difference (GBD) [9] and Top-hat transform (THT) [10] are used for microcalcification detection, but only in limited regions designated by radiologists or known calcification areas [10, 11]. Their activation in calcification-free areas (high false positives) limits screening utility.

A recent standard segmentation AI method for automatic pixel-level calcification detection across entire FFDM regions was reported [12, 13]. Compared to SP-based technologies, AI-based technology showed high sensitivity (over 95%) but had high false positives (PPV 3.24%), with over 94% of detected pixels not being actual calcifications. Recent SP-based refinement attempts to reduce AI false positives [14] decrease sensitivity, highlighting the need for new refinement methods.

Segmentation AI outputs binary probabilities between normality and abnormality. Generative AI provides image-domain values [15], such as virtual calcification-free FFDM images, offering specific pixel difference information beyond normal/abnormal classification. This yields informative features unavailable to segmentation AI, such as absolute calcification intensity, enabling more precise pixel-level detection.

**Figure 1.**
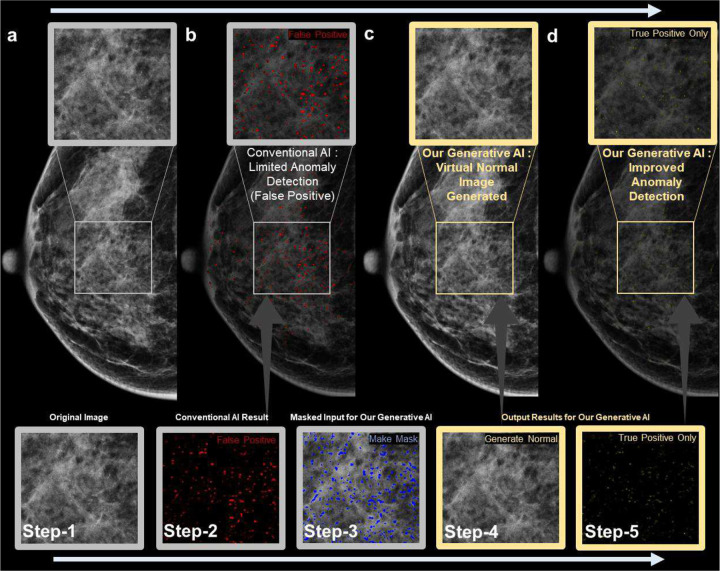
Steps for our generative AI technique: [Step-1] Use the original full-field digital mammography image as input, as shown in Figure 1(a). [Step-2] Conventional segmentation AI has high sensitivity but also high false positives (red pixels as shown in Figure 1(b)). [Step-3] Use the positive prediction result of conventional segmentation AI as a binary mask (blue) to obscure the corresponding area of the original image. [Step-4] Our generative AI replaces the masked area with virtual calcification-free pixels as its output, as shown in Figure 1(c). [Step-5] Calculating the difference between the output calcification-free pixels and the original leads to a more accurate calcification pixel extraction result (green pixels) than the conventional segmentation AI given in Step-2. This demonstrates that our generative AI can refine the pixel-level calcification detection probability map of conventional segmentation AI (updating results from Step-2 to Step-5) by significantly reducing false positives while preserving high sensitivity. More qualitative results of our generative AI are provided in Appendix (Figure A2).

**Figure 2.**
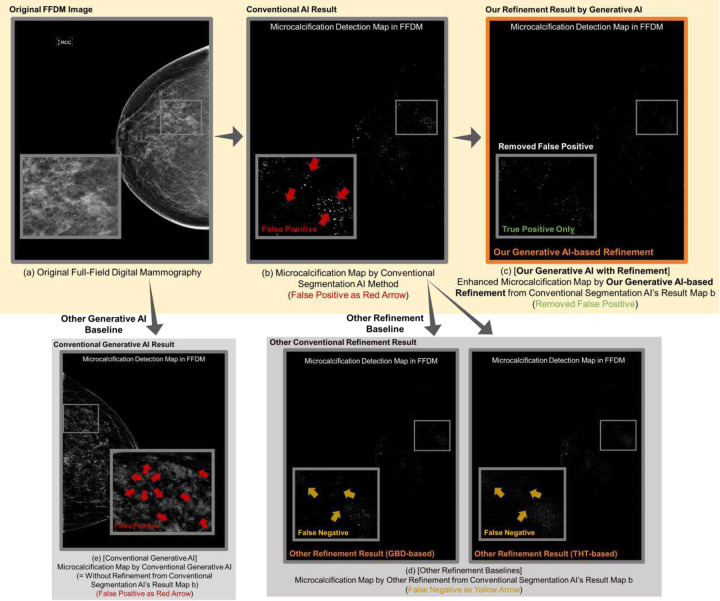
Summary of our study’s technical contributions: Existing AI technology for segmenting microcalcification in FFDM generates many false positives (b). Our generative AI refined this existing AI result to remove many false positives while maintaining high sensitivity (c). Our generative AI result (c) also resolves both the high false negative issues of other conventional refinement techniques (d) and the high false positive issues of existing generative AI techniques (e) for microcalcification detection in FFDM, as validated in Tables 1 (or 3) and 2, respectively.

Motivated by these advantages, our study aims to develop novel generative AI refinement technology (Figure 1, Table A1) to reduce segmentation AI’s false positives while maintaining its high sensitivity (Figure 2(c) and Table 1), addressing sensitivity decreases in conventional refinement methods (Figure 2(d) and Tables 1 and 3). Our advanced generative AI reduces false positives, improving PPV over 100 times (Figure 2(e) and Table 2) compared to current generative AI techniques [15].

## Backgrounds

### Conventional Methods for Pixel-level Calcification Detection in FFDM:

**Figure 3.**
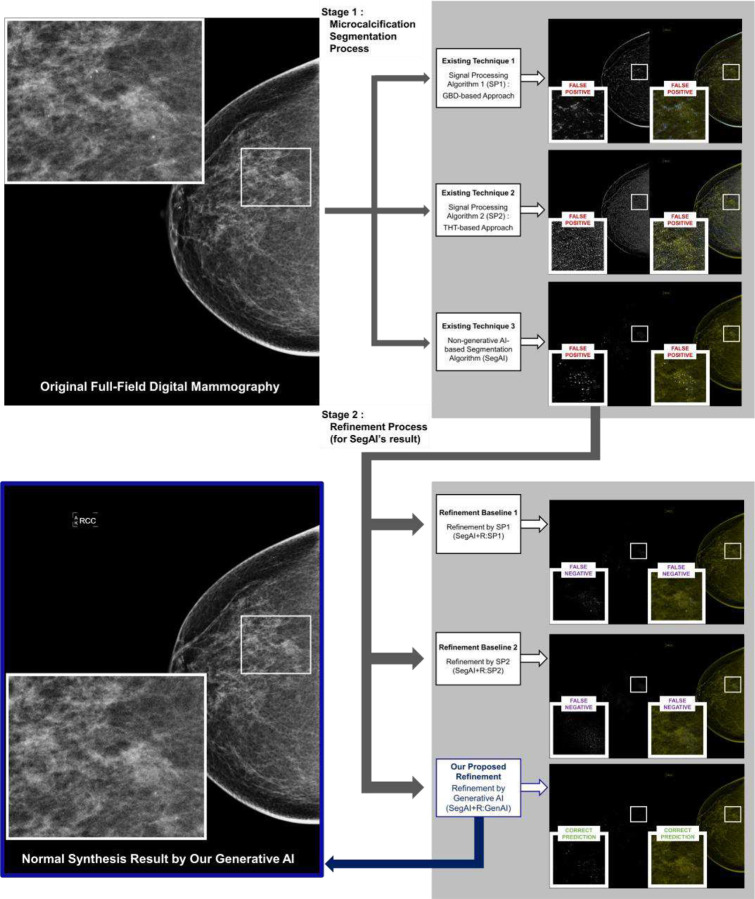
Study setup overview: This study compared six algorithms for microcalcification segmentation in full-field digital mammography image (FFDM). The three initial algorithms are: Gaussian blur difference (GBD)-based model (SP1), Top-hat transform (THT)-based model (SP2), conventional segmentation AI trained under supervised learning (SegAI). The first two are traditional signal processing and vision technologies, while the third is the latest general segmentation AI-based method. We observed that the AI-based technology (SegAI) has the highest sensitivity among the three but still has many false positives for PiCD-FFDM. To address SegAI’s false positives, we proposed three refinement algorithms for SegAI results: SP1-based refinement (SegAI+R:SP1), SP2-based refinement (SegAI+R:SP2), our proposed generative AI-based refinement (SegAI+R:GenAI). Our generative AI-based refinement effectively reduces SegAI’s false positives while maintaining its high sensitivity in pixel-level microcalcification detection (Table 1). Unlike other existing refinements (SegAI+R:SP1/2), our generative AI-based refinement shows superior sensitivity for pixel-level calcification detection (Table 1, Figures 5 and A4) and patient-level calcification detection (Table 3 and Figure 6) in entire FFDM images.

We implemented and compared six algorithms for pixel-level calcification detection in entire FFDM images (PiCD-FFDM), as shown in Figure 3: (a) Two conventional SP-based methods: GBD and THT (SP1 and SP2), (b) Conventional segmentation AI method (SegAI) [9, 10], (c) Two conventional SP-based refinement methods: GBD and THT (SegAI+R:SP1 and SegAI+R:SP2), (d) Our generative AI-based refinement method (SegAI+R:GenAI).

### Conventional SP-based methods (SP1 and SP2):

We optimized GBD and THT methods using average maps from multiple kernel sizes for calcification extraction, enabling detection of diverse-sized calcifications without manual parameter adjustments. Despite optimization, SP-based methods showed high sensitivity (96.33%) but extremely high false positive rates (PPV 0.09%, Table 1). This critical limitation occurs because calcification pixels are a small proportion of FFDM, yet these techniques activate in most calcification-free areas, misidentifying them as positive. This indicates SP-based methods are unsuitable for screening entire FFDM images, unlike application to specific radiologist-designated regions.

### Conventional segmentation AI method (SegAI):

A recent study [12, 13] demonstrated PiCD-FFDM using segmentation AI as an effective alternative to SP techniques. It reported a general segmentation network trained with supervised learning on pixel-level calcification annotations, outputting a binary calcification probability map for the entire FFDM. We implemented this method (SegAI) to validate its superiority over SP methods. Compared to SP-based methods (SP1 and SP2), SegAI significantly reduced false positives (PPV from 0.09% to 3.24%) while improving sensitivity (from 96.33% to 98%). However, it still showed a high false positive rate (low PPV of 3.24%, Table 1). To address this, we explored three refinement methods for SegAI.

### Conventional SP-based refinement methods (SegAI+R:SP1/2):

A recent study [12, 13] demonstrated PiCD-FFDM using segmentation AI as an effective alternative to SP techniques. It reported a general segmentation network trained with supervised learning on pixel-level calcification annotations, outputting a binary calcification probability map for the entire FFDM. We implemented this method (SegAI) to validate its superiority over SP methods. Compared to SP-based methods (SP1 and SP2), SegAI significantly reduced false positives (PPV from 0.09% to 3.24%) while improving sensitivity (from 96.33% to 98%). However, it still showed a high false positive rate (low PPV of 3.24%, Table 1). To address this, we explored three refinement methods for SegAI.

## Methods: Our Generative AI Methods for Pixel-level Calcification Detection in FFDM

**Figure 4.**
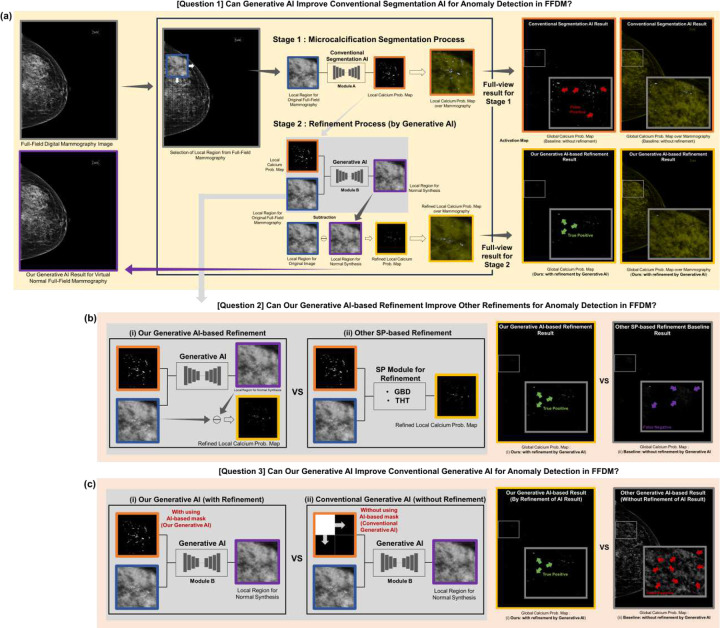
Methods of this study: Our method comprises two stages as shown in (a): In Stage 1, we use the conventional segmentation AI module to obtain a microcalcification segmentation map from the original full-field digital mammography image (FFDM) image. In Stage 2, we refine this result: using it as an input mask, our generative AI creates normal pixels for the masked area and calculates the difference with the original pixels to obtain a more accurate segmentation map, thus reducing false positives of the conventional segmentation AI result (Table 1). (b) Compared to other refinement methods (i.e., Gaussian blur difference (GBD)-based or Top-hat transform (THT)-based) that reduce false positives of conventional segmentation AI result but provide many false negatives, our proposed generative AI-based refinement method not only reduces false positives of conventional segmentation AI result but also reduces false negatives of other refinements (Tables 1 and 3). (c) To re-implement the existing latest generative AI technology, we use a random mask instead of the conventional segmentation AI result as the input mask for our generative AI in Stage 2. This baseline generative AI approach generates many false positives in the segmentation map than ours, demonstrating the superiority of our generative AI technology as the novel refinement tool for segmentation AI results in reducing their false positives while preserving high sensitivity (Table 2).

To reduce false positives while maintaining segmentation AI’s high sensitivity for PiCD-FFDM, we introduce generative AI to refine segmentation AI results (Figures 1, 2(c), 4(a)). Our model solves decreased sensitivity issues in other refinement technologies.

### Key process:

Our generative AI involves five steps (Figure 1): (1) Input random local FFDM region. (2) Obtain pixel-level microcalcification detection probability map using conventional segmentation AI [12, 13], providing high sensitivity but many false positives. (3) Use this map as blind mask input for generative AI. (4) Output calcification-free pixels for blind area, creating precise calcification-free version of original (Figures 1, A2). (5) Refine segmentation AI’s map by comparing original and output, reducing false positives (Figures 1, 2). This process iterates through each local region, combining results for final FFDM global outcome (details in Appendix, Figure A1).

### Key features of our generative AI compared to current segmentation AI methods:

Our generative AI mainly uses a two-stage approach (Figures 2(c), 3, 4(a)): obtaining a mask with conventional AI (Stage 1 in Figure 4(a)) and generating a refined anomaly map by inpainting this mask area and differentiating it from the original (Stage 2 in Figure 4(a)). This enables direct performance comparison between conventional segmentation AI (e.g., SegAI [9, 10]) and its refinement by our generative AI (SegAI+R:GenAI), as shown in Table 1. Existing segmentation AI (Stage 1) provides binary probability values for calcification presence in FFDM pixels but cannot determine actual calcification intensity. Our generative AI provides calcification intensity values by accurately generating calcification-free values for each FFDM pixel and calculating differences from original values (Figures 1, A2), a unique feature. This allows AI to recognize information beyond calcification presence, enabling more accurate pixel-level detection. Our generative AI demonstrates superiority by maintaining high sensitivity comparable to segmentation AI while improving PPV by more than twofold (Table 1 and Figure 2(c)).

### Key features of our generative AI compared to other refinement methods:

Our generative AI refines segmentation AI results to improve PiCD-FFDM performance (Figures 2(c), 4(a), Table 1), like other refinement methods. We compared it to traditional SP-based refinement methods to validate superiority (Figures 2(d), 4(b), Tables 1, 3). Figure 4(b) summarizes three refinement techniques: our generative AI-based (SegAI+R:GenAI) and two existing GBD/THT-based methods (SegAI+R:SP1/2). All use segmentation AI’s calcification anomaly prediction map as input, providing updated results. Other setups remained consistent across technologies.

Existing refinement methods (SegAI+R:SP1/2) detect calcification by calculating pixel differences based on point spreading functions, measuring intensity differences between calcification objects and surroundings. This method’s performance declines for smaller objects due to surrounding intensity bias (Table 3). Extracting absolute calcification object intensity is challenging, hindering robust detection across tissues and object densities.

Our generative AI non-parametrically provides accurate virtual calcification-free pixels, successfully extracting absolute calcification object intensity without size- and density-sensitive parameter optimization. This results in superior calcification pixel detection for various hard cases (Table 3).

### Key features of our generative AI compared to existing generative AI:

Our generative AI uses unsupervised learning, training on calcification-free images without annotations. It applies random masks, training to reproduce masked areas, ensuring true calcification-free pixel generation. Unlike other unsupervised generative AI methods with identical training phases, our testing phase differs (Figure 4(c)). Current methods use box-based random masks for each local area in testing, combining inpainted regions for a whole-area virtual image. Our method uses the external segmentation (i.e., non-generative) AI’s prediction results as new masks in testing (Figure 4(a)).

Table A1 shows current unsupervised generative AI models [11–16] were not designed to refine other AI results in testing. They apply random masks to entire target images (Figure 4(c)), generating whole-area virtual normal images, unnecessarily masking 100% of FFDM, limiting accuracy (Table 2 and Figure A3).

We found true calcification-positive pixels rarely exist outside segmentation AI masks (high sensitivity), with positive regions occupying <1.5% of FFDM (highly sparse as validated in Table 1). Our novel two-stage process (Figure 4(a)) uses non-generative AI results (e.g., general segmentation AI) as masks in testing, performing image transformation only within the mask. This minimizes unnecessary transformations in exterior regions (i.e., true negative regions) and effectively addresses false positive issues of current generative AI solvers, validated in Table 2 and Figure A3.

## ResuIts

This study performed all algorithms on the external public InBreast dataset (126 FFDM mammograms) [17]. Details for dataset and test setup of this study are provided in Appendix.

### Setup for dataset:

We utilized three datasets; European Radiology public dataset (81 FFDM mammograms) [18] with pixel-level calcification annotations for training segmentation AI (SegAI), referencing [9, 10]. Our institutional dataset (1121 FFDM mammograms) annotated for calcification-free regions to train our generative AI, referencing [16, 19], whose training process is detailed in Appendix. External InBreast public dataset (126 FFDM mammograms) [17] for algorithm validation.

### Test setup for pixel-level calcification-anomaly detection in FFDM:

We evaluated PiCD-FFDM performance, comparing our generative AI with five existing non-generative algorithms (Figure 3) and a baseline generative model (Figure 4(c)). Results are in Tables 1 and 2. All methods use 126 FFDM test images from InBreast dataset [17] as input, generating calcification anomaly probability maps.

For each FFDM image, we averaged sensitivity and PPV values of pixel-level calcification binary classification results, then calculated image-level average, standard deviation, and *p*-value. Activation rate is the ratio of predicted calcification-positive pixel area within breast region, averaged per image. We also calculated and averaged Dice coefficient per image.

### Test setup for patient-level calcification-anomaly detection in FFDM:

We assessed patient-level calcification-anomaly detection (PaCD-FFDM) by classifying patients as abnormal if FFDM output contained at least one true calcification object. Patient-level binary classification accuracy is shown in Figure 6 and Table 3, comparing our generative AI-based refinement method with two refinement techniques. To verify our method’s superiority in challenging cases, we evaluated performance under three conditions: (a) Calcification objects smaller than a specific size (Figure 6(a)), (b) Excluded cases with exterior tissue density below threshold (Figure 6(b)), (c) Excluded cases with interior calcification density above threshold (Figure 6(c)). These represent difficult conditions: smaller size, higher surrounding tissue density, and lower calcification density, respectively. Patients without remaining true calcifications after exclusions were omitted and all methods were consistently applied with same number of patients for fair comparison for each condition. These evaluations demonstrated our generative AI method’s superior performance in detecting calcifications under difficult conditions.

### Test result for pixel-level calcification-anomaly detection in FFDM:

**Figure 5.**
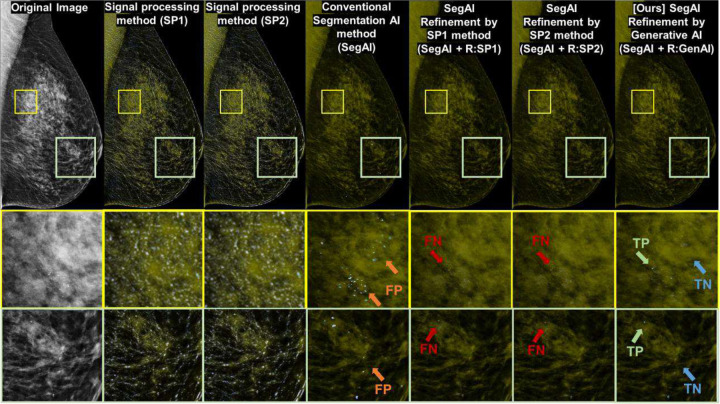
Qualitative performance comparison between algorithms (i.e., qualitative results for Table 2 and Figure 4): Our generative AI method (SegAI+R:GenAI) achieves better results (reduced false positives) than the conventional segmentation AI method (SegAI). It also shows improved results (reduced false negatives) compared to other conventional refinement methods (SegAI+R:SP1 or SegAI+R:S2). More details are provided in Appendix (Figure A4).

Table 1 and Figure 5 (or A4) compare the PiCD-FFDM performance of our generative AI with five other methods as shown in Figure 2, including conventional segmentation AI (SegAI) and other refinements of SegAI (SegAI+R). Compared to segmentation AI (SegAI in Figure 4(a)), our generative AI (SegAI+R:GenAI) maintains high sensitivity over 95% and significantly improves PPV and activation rate by 2.25 times (from 3.24% to 7.30%; *p*<0.05) and 2.83 times (from 1.22% to 0.43%; *p*<0.05), respectively. Compared to other refinements (SegAI+R:SP1/2), our generative AI (SegAI+R:GenAI) has the highest sensitivity (95.85%; *p*<0.05) and the lowest activation rate (0.43%; *p*<0.05).

Table 2 and Figure A3 compare the PiCD-FFDM performance of our generative AI with the latest generative AI (Figure 4(c)). Both methods show high sensitivity (over 95%), but the existing method has a high false positive rate (i.e., low PPV 0.05% and high activation rate 76.50%). Our method significantly improves these metrics, increasing PPV by 146 times (from 0.05% to 7.30%; *p*<0.05) and reducing the activation rate by 178 times (from 76.50% to 0.43%; *p*<0.05), while preserving high sensitivity over 95%.

### Test result for patient-level calcification-anomaly detection in FFDM:

**Figure 6.**
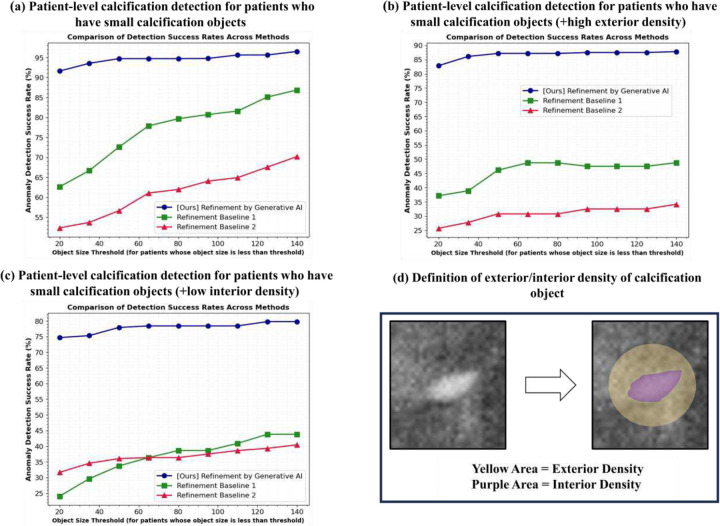
Comparison of patient-level microcalcification detection performance between refinement algorithms: This comparison assesses the superiority of our proposed generative AI refinement over signal processing refinements in microcalcification detection. (a) shows performance for patients with microcalcifications smaller than the x-axis value. (b) depicts results for patients with high exterior density microcalcifications. (c) illustrates performance for patients with low interior density microcalcifications. Results demonstrate that our generative AI outperforms other refinements in all scenarios, with a notably larger performance difference for patients with small objects (a), high exterior density (b), and low interior density (c) microcalcifications. Detailed test setup and interpretation are provided in Appendix.

To further validate the superiority of our generative AI as a refinement compared to other refinement methods, we conducted a comparative analysis based on calcification size and density. The patient-level calcification detection (PaCD-FFDM) results are presented in Figure 6 and Table 3. As shown in Table 3, our generative AI-based refinement (SegAI+R:GenAI) significantly outperforms other refinement methods (SegAI+R:SP1/2) with *p*<0.05. The improvements are as follows:

- For patients with small calcifications (i.e., less than 50 pixels), our generative AI improves the detection error rates by 5.17 times (from 27.43% (31/113) to 5.31% (6/113)).
- For patients with small calcifications and high exterior density, our model enhances the detection error rates by 4.20 times (from 53.85% (21/39) to 12.82% (5/39)).
- For patients with small calcifications and low interior density, our model reduces the error rates by 2.89 times (from 63.95% (55/86) to 22.09% (19/86)).

### Accuracy test of virtual normal samples from our generative AI:

Our proposed generative AI takes a 512×512 pixel original FFDM patch as input and generates a normal version of the same size. To measure the accuracy of our AI in creating normal images, we conducted an analysis shown in Table 4. Using the InBreast dataset’s 126 FFDM images, we generated normal FFDM versions (Figures 1 and A2) by dividing each FFDM into overlapping 512×512 pixel patches and synthesizing the outputs from our generative AI (Details for method were given in Figure A1). We then compared the calcification-free virtual normal FFDM images with the original FFDMs using pixel annotations to define normal and abnormal regions. The Global-level Test in Table 4 compared the original FFDM with the AI-generated normal FFDM, while the Local-level Test compared each 512×512 patch to its corresponding original. We used three metrics for these comparisons:

- PSNR (or RMSE) - Measures the average difference in pixel values, with higher PSNR (or lower RMSE) indicating smaller differences.
- Error Ratio - Defines errors where the pixel difference exceeds 1, calculating the percentage of error-free regions at the image level. This shows how accurately our AI predicts normal pixels in normal regions (99.9%) and abnormal pixels in abnormal regions (90.5% or 91.6%).

Experimental results showed that our generative AI accurately identified normal pixel regions with a PSNR of over 50 and an error rate of 0.1% at both the global (FFDM) and local (patch) levels. This indicates minimal false negatives and no unnecessary artifacts. Importantly, our AI changed over 90% of actual calcification pixels in abnormal regions to normal pixels. The average pixel change in abnormal regions was 373.5 (local) or 512.4 (global) DICOM standard pixels, over 60 times the change in normal regions

## Discussion

Our generative AI significantly improves pixel-level calcification detection over segmentation AI (i.e., non-generative AI). Segmentation AI has high sensitivity but many false positives. Our generative AI refinement maintains sensitivity while halving false detections. It learns from calcification-free images via unsupervised anomaly detection. Unlike other methods using random masks causing distortion, ours applies them only in training. During inference, it uses deterministic masks from segmentation AI to refine detection maps, targeting calcification candidates. This reduces distortion and improves PPV over 100-fold versus existing generative AI. Our method enables automatic pixel-level calcification detection in FFDM, outperforming three conventional approaches (i.e., segmentation AI, its refinement, and unsupervised generative AI) in both sensitivity and PPV. Particularly, ours achieves over 85% detection for small calcifications in dense tissues, where existing refinement methods had under 50% success (over 2.5-fold error reduction). This shows the potential of generative AI for clinical FFDM screening of abnormalities across patients.

Recent studies [11–16] show unsupervised generative AI models in medicine can detect anomalies using only normal data. This overcomes generalization issues from diverse anomaly patterns that can bias networks if used in training. Our generative AI learns from normal (calcification-free) data to detect various anomalies, with potential beyond calcifications. Most medical unsupervised generative AI models focus on brain MRI tumor detection, a relatively easy task with tumors exceeding 5% of brain volume and high human detection rates. Detecting microcalcifications in FFDM is more challenging, occupying less than 0.5% of breast images (Table 1), especially difficult in dense tissue with small objects where human detection is low. This represents a significant screening challenge. Our study first demonstrates superior unsupervised generative AI performance in this area compared to other technologies [11–16] (Tables 2 and 3), suggesting its potential for accurate anomaly localization in various radiological challenges.

In mammography, detecting small calcifications in dense breast tissue is challenging, with critical clinical implications. Sensitivity for microcalcifications in dense breasts can be as low as 50% (Table 3), leading to missed diagnoses and delayed treatment [4]. High false-positive rates cause unnecessary biopsies and patient anxiety [20]. Traditional signal processing techniques struggle with variations in resolution and tissue density [21], necessitating more robust approaches. Our novel generative model addresses these limitations (Figure 6 and Table 3), using a pure AI approach without signal processing. This nonparametric method accurately detects calcifications across varying object resolutions and tissue densities. The AI model, trained unsupervised using only normal data, avoids overfitting to specific calcification information [22], enabling reliable detection of diverse calcifications to offer a robust clinical solution.

Our AI technology halves false positives while maintaining over 95% sensitivity compared to the latest segmentation AI methods, significantly impacting mammography calcification screening. This reduction could minimize unnecessary biopsies, reduce patient anxiety, and improve diagnostic efficiency, enhancing patient care [23, 24]. High sensitivity ensures the detection of more calcifications, potentially enabling earlier breast cancer detection even within high tissue density, including invasive cases from ductal carcinoma in situ [25]. Improved calcification detection accuracy may help identify high-risk patients needing closer monitoring or preventive measures [26]. These advancements highlight our AI technology’s clinical value in enhancing early breast cancer detection and optimizing patient management.

Our generative AI accurately provides pixel-level matched virtual normal images for FFDM, a previously unattainable capability [27]. This novel reference enables radiologists to perform analyses impossible before, like comparing current images with initial records, even without pixel-level matching [28]. This extends beyond screening, allowing clinical applications such as prognosis analysis (e.g., invasive breast cancer prediction). By providing a new precise and patient-specific reference as normal, our AI could facilitate early detection of calcifications with subtle existence, potentially improving breast cancer management outcomes [29]. The technology also aids in the longitudinal assessment of treatment response, enabling personalized therapy adjustments and enhancing overall patient care.

## Conclusion

Our generative AI model significantly reduces false positives and enhances sensitivity in detecting calcifications on mammography, particularly effective in complex cases involving small calcifications of varying densities. Our generative AI model generates precise virtual normal mammograms, which when aligned with the original abnormal images, facilitate pixel-level difference analysis, assisting radiologists in the early detection of breast cancer.

## Supplementary Files

This is a list of supplementary files associated with this preprint. Click to download.

- Supplementarymaterial.docx

## Figures and Tables

**Table 1.** Performance comparison between methods for pixel-level calcifcation anomaly detection throughout FFDM (PiCD-FFDM): Table 1 demonstrates our generative AI’s ability to outperform traditional segmentation AI in anomaly detection (Question 1, Figure 4). Unlike existing inpainting-based generative AI using random masks, our method refines segmentation AI masks [11–16], significantly reducing false positives. Compared to segmentation AI (SegAI, Table 1; Stage 1, Figure 4(a)), our generative AI (SegAI+R:GenAI, Table 1; Stage 2, Figure 4(a)) maintains high sensitivity (>95%) and improves PPV 2.3 times from 3.24% to 7.30% (p<0.05). This is the first demonstration of generative AI outperforming traditional segmentation AI. Table 1 also addresses whether generative AI outperforms traditional refinement techniques (Question 2, Figure 4). Our generative AI-based refinement (SegAI+R:GenAI) shows the highest sensitivity (95.83%) compared to traditional refinement techniques (SegAI+R:SP1 and SegAI+R:SP2). A detailed performance comparison is in Table 3. Qualitative results for Table 1 were given in Figures 5 and A4, with test setup and interpretation details in Appendix.

Method \ Performance Metric (%)	Sensitivity	PPV/Precision	DICE	Activation Area (True Activation Area)	Create a virtual normal?
Signal Processing Algorithm 1 without Refinement (SP1)	96.33 ± 3.43	0.05 ± 0.11*	0.09 ± 0.22*	78.48 ± 4.18* (0.034 ± 0.075)	No
Signal Processing Algorithm 2 without Refinement (SP2)	61.12 ± 12.64*	0.09 ± 0.18*	0.17 ± 0.36*	22.34 ± 2.36* (0.019 ± 0.042)	No
**Non-genAI Algorithm without Refinement (SegAI)**	**98.00 ± 1.85** *	**3.24 ± 7.40** *	**5.47 ± 10.86** *	1.22 ± 0.38* **(0.034 ± 0.075)**	No
Refinement of SegAI by SP1 (SegAI+R:SP1)	92.42 ± 7.04*	8.21 ± 11.06	13.39 ± 16.09	0.66 ± 0.68* (0.034 ± 0.076)	No
Refinement of SegAI by SP2 (SegAI+R:SP2)	55.98 ± 13.56*	1.93 ± 2.87*	3.46 ± 4.72*	1.28 ± 1.49* (0.017 ± 0.039)	No
**[Ours] Refinement of SegAI by GenAI (SegAI+R:GenAI)**	**95.83 ± 4.49**	**7.30 ± 13.50**	**11.28 ± 17.17**	**0.43 ± 0.16 (0.033 ± 0.073)**	**Yes**

* indicates that the target algorithm’s performance differs from that of our generative AI-based refinement method (i.e., SegAI+R:GenAI) under statistical significance *p* <0.05

**Table 2.** Performance comparison for PiCD-FFDM between our (SegAI+R:GenAI) and current generative AI methods: Table 2 addresses whether our generative AI outperforms existing methods (Question 3, Figure 4). We introduced a novel AI technique generating normal patient images. Unlike recent generative AI methods [11–16] not using SL-based segmentation masks (Table A1), our approach uses a two-step process utilizing and updating the SL-based mask (Figure 4(a)). This allows our generative AI to improve PPV for pixel-level microcalcification detection by over 146 times in FFDM, compared to methods not using SL-based masks. It demonstrates superiority with PPV improvement from 0.05% to 7.30% (p<0.05), while maintaining high sensitivity above 95% (Table 2). This is the first demonstration of significantly improved generative AI performance via novel application as refinement for SL-based mask. Qualitative results are in Figure A3, with test setup and interpretation details in Appendix.

Method \ Performance Metric (%)	Sensitivity	PPV/Precision	DICE	Activation Area (True Activation Area)
Our Generative AI (i.e., With using AI-based Mask)	**95.83 ± 4.49**	**7.30 ± 13.50**	**11.28 ± 17.17**	**0.43 ± 0.16 (0.033 ± 0.073)**
Baseline Generative AI (i.e., Without using AI-based Mask)	99.17 ± 1.75*	0.05 ± 0.13*	0.10 ± 0.26*	76.52 ± 5.62* (0.035 ± 0.078)

* indicates that the target algorithm’s performance differs from that of our generative AI-based refinement method (i.e., Top row result: SegAI+R:GenAI) under statistical significance *p* <0.05

**Table 3.** Patient-level calcification anomaly detection error rate (PaCD-FFDM) for patients who have small cal. objects (all object sizes under 50 pixels = negative value of third point on x-axis in Fig. 6): Table 3 compares detection error rates for PaCD-FFDM at x = 50 in Figure 6. Our generative AI outperforms other refinements, especially for patients with small calcifications (pixels ≤ 50) by more than 5.17 times (from 27.43% to 5.31%), with small calcifications and high-exterior density by more than 4.20 times (from 53.85% to 12.82%), and with small calcifications and low-interior density by more than 2.89 times (from 63.95% to 22.09%).

Refinement Method \ Setup	Fig. 6(a)	Fig. 6(b)	Fig. 6(c)
Refinement by SP1 (SegAI+R:SP1)	27.43 ± 44.62*	53.85 ± 49.85*	66.28 ± 47.28*
Refinement by SP2 (SegAI+R:SP2)	43.36 ± 49.56*	69.23 ± 46.15*	63.95 ± 48.01*
**[Ours] Refinement by GenAI (SegAI+R:GenAI)**	**5.31 ± 22.42**	**12.82 ± 33.43**	**22.09 ± 41.49**

* *p* <0.05 by performance comparision with our algorithm, SegAI+R:GenAI

**Table 4.** Accuracy of Virtual Normal Output Samples from Our Generative AI: While we lack true normal FFDM data for our evaluation, the results in Table 4 demonstrate that our AI effectively alters only the abnormal pixels without affecting the normal ones. The accuracy of our AI’s normal generation is further evidenced by its higher PPV (over twice that of segmentation AI (SegAI)) and the same high sensitivity (over 95%) when detecting calcification pixels, as shown in Table 1.

[Local-level Test] Class \ Performance Metric	PSNR (dB)	RMSE	Error Ratio (%)
Ground Truth Normal Region	51.3	6.3	0.1
Ground Truth Abnormal Region	17.9	373.5	90.5
[Global-level Test] Class \ Performance Metric	PSNR (dB)	RMSE	Error Ratio (%)
Ground Truth Normal Region	51.8	7.6	0.1
Ground Truth Abnormal Region	15.7	512.4	91.6

## Data Availability

Data are available at European Radiology [18] and InBreast [17] public datasets, respectively : https://zenodo.org/records/5036062#.Y7XQB3bMLyY https://www.kaggle.com/datasets/ramanathansp20/inbreast-dataset. While the source code is not provided, all experimental procedures and implementation details are thoroughly described in the Supplementary Material to ensure full reproducibility.

## References

[1] Tabár L., , The Swedish Two-County Trial twenty years later: updated mortality results and new insights from long-term follow-up. Radiologic Clinics of North America, 2000. 38(4): p. 625–651. 10943268 10.1016/s0033-8389(05)70191-3

[2] Virnig B.A., , Ductal carcinoma in situ: risk factors and impact of screening. Journal of the National Cancer Institute Monographs, 2010. 2010(41): p. 113–116. 20956813 10.1093/jncimonographs/lgq024 PMC5161075

[3] Weigelt B., Peterse J.L., and Veer L.J. Van’t, Breast cancer metastasis: markers and models. Nature reviews cancer, 2005. 5(8): p. 591–602. 16056258 10.1038/nrc1670

[4] Kolb T.M., Lichy J., and Newhouse J.H., Comparison of the performance of screening mammography, physical examination, and breast US and evaluation of factors that influence them: an analysis of 27,825 patient evaluations. Radiology, 2002. 225(1): p. 165–175. 12355001 10.1148/radiol.2251011667

[5] Berry D.A., , Effect of screening and adjuvant therapy on mortality from breast cancer. New England Journal of Medicine, 2005. 353(17): p. 1784–1792. 16251534 10.1056/NEJMoa050518

[6] Birdwell R.L., , Mammographic characteristics of 115 missed cancers later detected with screening mammography and the potential utility of computer-aided detection. Radiology, 2001. 219(1): p. 192–202. 11274556 10.1148/radiology.219.1.r01ap16192

[7] Pisano E.D., , Diagnostic performance of digital versus film mammography for breast-cancer screening. New England Journal of Medicine, 2005. 353(17): p. 1773–1783. 16169887 10.1056/NEJMoa052911

[8] Brewer N.T., , Meta-analysis of the relationship between risk perception and health behavior: the example of vaccination. Health psychology, 2007. 26(2): p. 136. 17385964 10.1037/0278-6133.26.2.136

[9] Gerbasi A., , DeepMiCa: Automatic segmentation and classification of breast MIcroCAlcifications from mammograms. Computer Methods and Programs in Biomedicine, 2023. 235: p. 107483. 37030174 10.1016/j.cmpb.2023.107483

[10] Wu X., Hong D., and Chanussot J., UIU-Net: U-Net in U-Net for infrared small object detection. IEEE Transactions on Image Processing, 2022. 32: p. 364–376. 37015404 10.1109/TIP.2022.3228497

[11] Behrendt F., , Patched diffusion models for unsupervised anomaly detection in brain MRI. arXiv 2023. arXiv preprint arXiv:2303.03758.

[12] Bercea C.I., , Diffusion Models with Implicit Guidance for Medical Anomaly Detection. arXiv preprint arXiv:2403.08464, 2024.

[13] Iqbal H., Unsupervised anomaly detection in medical images using masked diffusion model*. in* International Workshop on Machine Learning in Medical Imaging. 2023. Springer.

[14] Wolleb J., , Binary Noise for Binary Tasks: Masked Bernoulli Diffusion for Unsupervised Anomaly Detection. arXiv preprint arXiv:2403.11667, 2024.

[15] Xu R., Wang Y., and Du B., MAEDiff: Masked Autoencoder-enhanced Diffusion Models for Unsupervised Anomaly Detection in Brain Images. arXiv preprint arXiv:2401.10561, 2024.

[16] He Y., , Disorder-Free Data Are All You Need—Inverse Supervised Learning for Broad-Spectrum Head Disorder Detection. NEJM AI, 2024. 1(4): p. AIoa2300137.

[17] Moreira I.C., , Inbreast: toward a full-field digital mammographic database. Academic radiology, 2012. 19(2): p. 236–248. 22078258 10.1016/j.acra.2011.09.014

[18] Loizidou K., , Digital subtraction of temporally sequential mammograms for improved detection and classification of microcalcifications. European radiology experimental, 2021. 5: p. 1–12. 34519867 10.1186/s41747-021-00238-w PMC8440760

[19] Li W., Mat: Mask-aware transformer for large hole image inpainting*. in* Proceedings of the IEEE/CVF conference on computer vision and pattern recognition. 2022.

[20] Nelson H.D., , Harms of breast cancer screening: systematic review to update the 2009 US Preventive Services Task Force recommendation. Annals of internal medicine, 2016. 164(4): p. 256–267. 26756737 10.7326/M15-0970

[21] Giger M.L., Karssemeijer N., and Schnabel J.A., Breast image analysis for risk assessment, detection, diagnosis, and treatment of cancer. Annual review of biomedical engineering, 2013. 15(1): p. 327–357. 10.1146/annurev-bioeng-071812-152416 23683087

[22] Schlegl T., Unsupervised anomaly detection with generative adversarial networks to guide marker discovery*. in* International conference on information processing in medical imaging. 2017. Springer.

[23] Brewer N.T., Salz T., and Lillie S.E., Systematic review: the long-term effects of false-positive mammograms. Annals of internal medicine, 2007. 146(7): p. 502–510. 17404352 10.7326/0003-4819-146-7-200704030-00006

[24] Pedemonte S., , A Semiautonomous Deep Learning System to Reduce False Positives in Screening Mammography. Radiology: Artificial Intelligence, 2024. 6(3): p. e230033. 38597785 10.1148/ryai.230033 PMC11140506

[25] Stomper P.C., , Mammographic predictors of the presence and size of invasive carcinomas associated with malignant microcalcification lesions without a mass. American Journal of Roentgenology, 2003. 181(6): p. 1679–1684. 14627596 10.2214/ajr.181.6.1811679

[26] Kerlikowske K., , Comparative effectiveness of digital versus film-screen mammography in community practice in the United States: a cohort study. Annals of internal medicine, 2011. 155(8): p. 493–502. 22007043 10.7326/0003-4819-155-8-201110180-00005 PMC3726800

[27] Gur D., , Dose reduction in digital breast tomosynthesis (DBT) screening using synthetically reconstructed projection images: an observer performance study. Academic radiology, 2012. 19(2): p. 166–171. 22098941 10.1016/j.acra.2011.10.003 PMC3251730

[28] Pinto Pereira S.M., , Automated registration of diagnostic to prediagnostic x-ray mammograms: Evaluation and comparison to radiologists’ accuracy. Medical physics, 2010. 37(9): p. 4530–4539. 20964170 10.1118/1.3457470

[29] Kerlikowske K., , Identifying women with dense breasts at high risk for interval cancer: a cohort study. Annals of internal medicine, 2015. 162(10): p. 673–681. 25984843 10.7326/M14-1465 PMC4443857

